# Blumgart anastomosis with polyglycolic acid felt reduces the incidence of pancreatic fistula after pancreaticoduodenectomy: A propensity score analysis

**DOI:** 10.1002/ags3.12598

**Published:** 2022-06-30

**Authors:** Yasuyuki Fukami, Takuya Saito, Takaaki Osawa, Shunichiro Komatsu, Tsuyoshi Sano

**Affiliations:** ^1^ Division of Gastroenterological Surgery, Department of Surgery Aichi Medical University Nagakute Japan

**Keywords:** Blumgart anastomosis, pancreatic fistula, pancreaticoduodenectomy, pancreaticojejunostomy, polyglycolic acid

## Abstract

Polyglycolic acid (PGA) felt has been used to prevent clinically relevant postoperative pancreatic fistula (CR‐POPF) after pancreaticoduodenectomy (PD). However, there has been no related research on Blumgart anastomosis. Therefore, this study aimed to investigate the practical significance of Blumgart anastomosis with our new method involving PGA felt to prevent CR‐POPF following PD. Data from 133 consecutive patients who underwent Blumgart anastomosis during PD between June 2015 and November 2021 were reviewed. We applied PGA felt to 35 of these patients starting from July 2020. Surgical outcomes were analyzed by propensity score matching. Thirty‐five (35.7%) of the 98 patients in the without‐PGA group were matched with an equal number from the with‐PGA group after adjusting for covariates. CR‐POPF was found in 17 patients (49%) in the without‐PGA group and two patients (6%) in the with‐PGA group (*P* < .001). The multivariate analysis results indicated that male sex, pancreatic duct size <3 mm, soft pancreatic texture, and nonuse of PGA were independently associated with CR‐POPF after PD. In conclusion, Blumgart anastomosis with our new penetrating method for PGA felt reduces the incidence of CR‐POPF after PD.

## Graphical abstract

This study aimed to investigate the practical significance of Blumgart anastomosis with our new method involving polyglycolic acid (PGA) felt to prevent clinically relevant postoperative pancreatic fistula (CR‐POPF) following pancreaticoduodenectomy (PD). In this study, our new method with the PGA felt reduces the incidence CR‐POPF after PD.

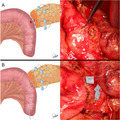

## 1 INTRODUCTION

Pancreaticoduodenectomy (PD) is a complex surgical procedure that is indicated for pancreatic‐head and periampullary tumors. Because of its high complication rate, PD is recommended to be performed at a high‐volume center.^1^ Postoperative pancreatic fistula (POPF) remains one of the most common and intractable complications after PD. Despite modifications in surgical techniques and perioperative patient care to prevent POPF, the incidence of POPF has been reported to range from 3% to 50%, even at high‐volume centers.^2^, ^3^ The POPF grading scale was defined by the International Study Group of Pancreatic Surgery (ISGPS), and grade B or C POPF is regarded as clinically relevant POPF (CR‐POPF). CR‐POPF can cause further life‐threatening complications (eg, intraabdominal abscess, intraabdominal bleeding, and septicemia) and lead to prolonged hospital stays and increased healthcare costs.

Blumgart anastomosis is the tension‐free approximation of the jejunum to the pancreatic stump by transfixing mattress sutures,^4^ which has been modified in various institutions with good outcomes.^5^ Therefore, Blumgart anastomosis has been adopted by many institutions.

Polyglycolic acid (PGA) felt is a bioabsorbable recombinant membrane made of a synthetic polymer with a cellulose‐like structure and has been widely used to reinforce tissues such as the lung and bronchi in thoracic surgery. Recently, PGA felt has been used to prevent POPF in pancreatic surgery.^6^, ^7^, ^8^, ^9^, ^10^ Regarding PD, some retrospective studies indicated that the incidence of CR‐POPF was lower in patients with PGA than in those without PGA.^7^, ^8^ In contrast, some other studies did not show a significant difference in the incidence of CR‐POPF between patients with and without PGA.^9^, ^10^ In addition, the pancreaticojejunostomy of these studies was not performed by Blumgart anastomosis, and the PGA felt was wrapped around the anastomotic site as a method of reinforcement. Recently, we developed a new method for the PGA felt to adhere without wrapping during Blumgart anastomosis. Therefore, this study aimed to investigate the practical significance of Blumgart anastomosis with PGA felt to prevent CR‐POPF following PD.

## 2 PATIENTS AND METHODS

Between June 2015 and November 2021, 133 consecutive patients who underwent Blumgart anastomosis during PD at our institution were enrolled in the study. Because the diameter of the main pancreatic duct was extremely small, patients undergoing dunking anastomosis (n = 1) were excluded. Patients undergoing hepatopancreaticoduodenectomy (n = 11) were also excluded. Between June 2015 and June 2020, 98 patients underwent Blumgart anastomosis without PGA, while the remaining 35 patients underwent the PGA felt penetrating method between July 2020 and November 2021. During this study period, one expert surgeon (T.S.) participated in 128 of 133 surgeries. This surgeon performed PJ anastomosis in 54 of 98 patients in the with‐PGA group and 23 of 35 patients in the without‐PGA group. The demographics, clinical characteristics, operative details, and postoperative outcomes of patients with and without CR‐POPF were retrospectively analyzed. All patients provided written informed consent before undergoing therapy. This study was approved by the Institutional Review Board of our institution (No. 2021‐149) and was performed in accordance with the 1964 Declaration of Helsinki and its later amendments or comparable ethical standards.

### 2.1 Surgical procedure

After review by a multidisciplinary board, all pancreatic disease cases were assessed by pancreatic surgeons to determine resectability and the most appropriate surgical procedure. The subtotal stomach‐preserving method was the standard procedure for PD. In patients with malignant disease, lymph nodes were dissected at the hepatoduodenal ligament, around the common hepatic artery, around the superior mesenteric artery (SMA), and around the pancreatic head. Transection of the pancreatic parenchyma was performed with an electric scalpel. A modified Child method, with duct‐to‐mucosa pancreatojejunostomy, was chosen for organ reconstruction in all cases. The modified Blumgart mattress suture was performed for pancreatic remnant reconstruction.^5^, ^11^ In all cases, a 4‐Fr polyethylene tube was placed through the pancreatojejunal anastomotic site as an external stent. Three silastic flexible drains were routinely placed adjacent to the anastomosis and at both the cranial and caudal sites of the pancreatojejunostomy and choledochojejunostomy.

**FIGURE 1:**
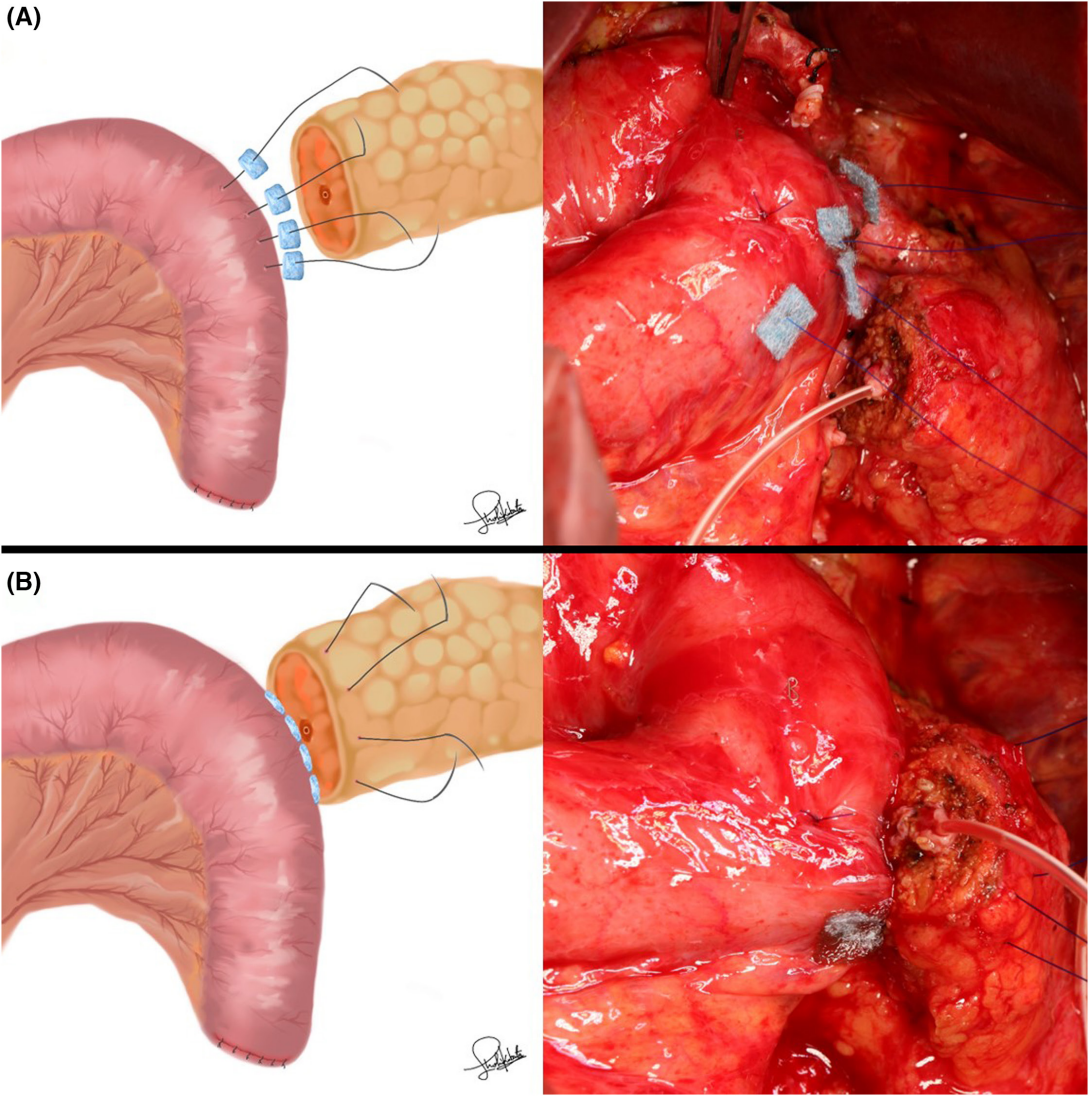
(A) After the seromuscular layer of the jejunum was threaded through using straight needles with 4‐0 monofilament nonabsorbable sutures, 7 × 7 mm PGA felts were penetrated by each needle to reinforce the dorsal side. (B) The pancreatic parenchyma was sutured penetratingly

**FIGURE 2:**
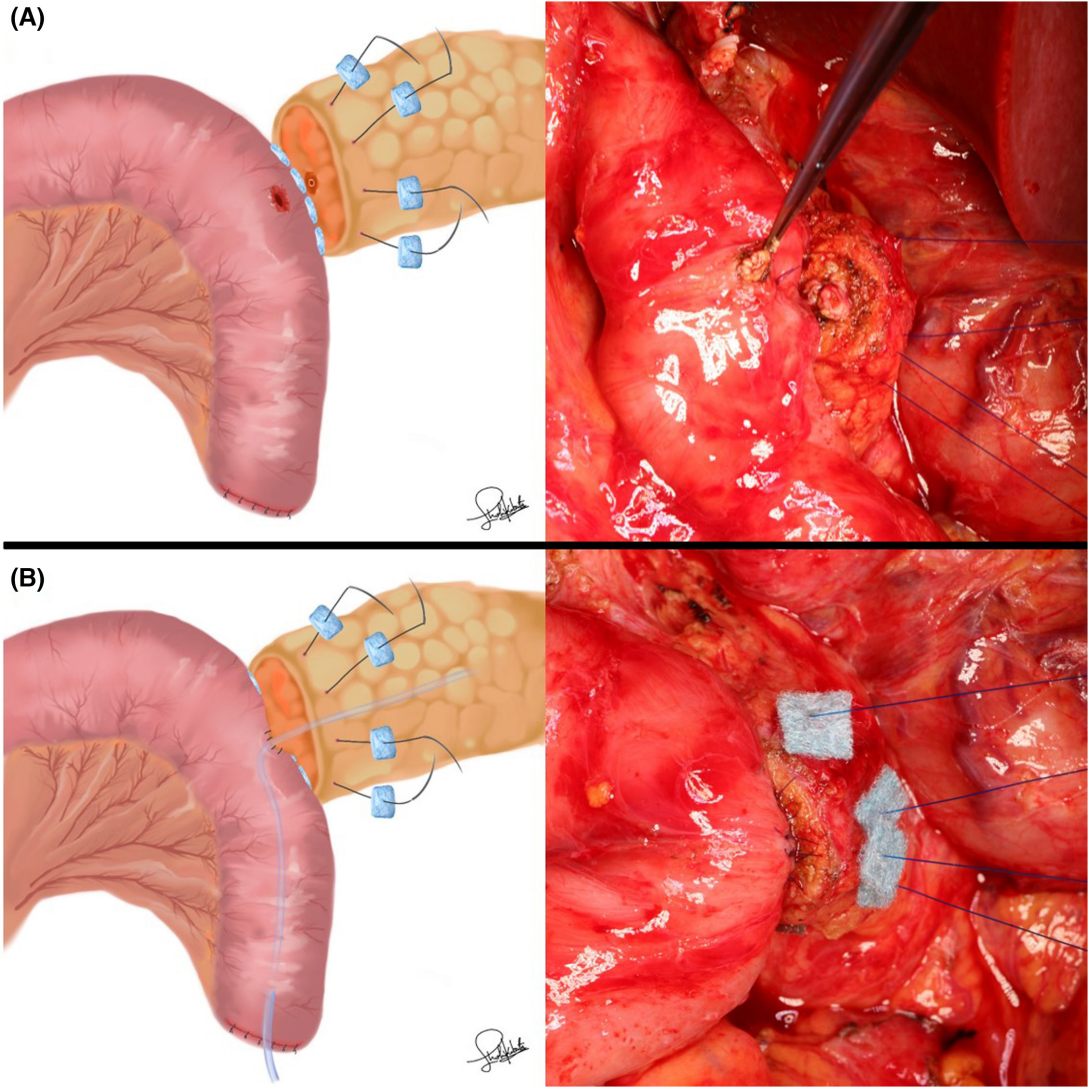
(A) For reinforcement of the ventral side, 7 × 7 mm PGA felts were penetrated by each needle. (B) Anastomosis between the main pancreatic duct and the jejunal wall was performed with 6‐8 interrupted 5‐0 monofilament absorbable sutures

**FIGURE 3:**
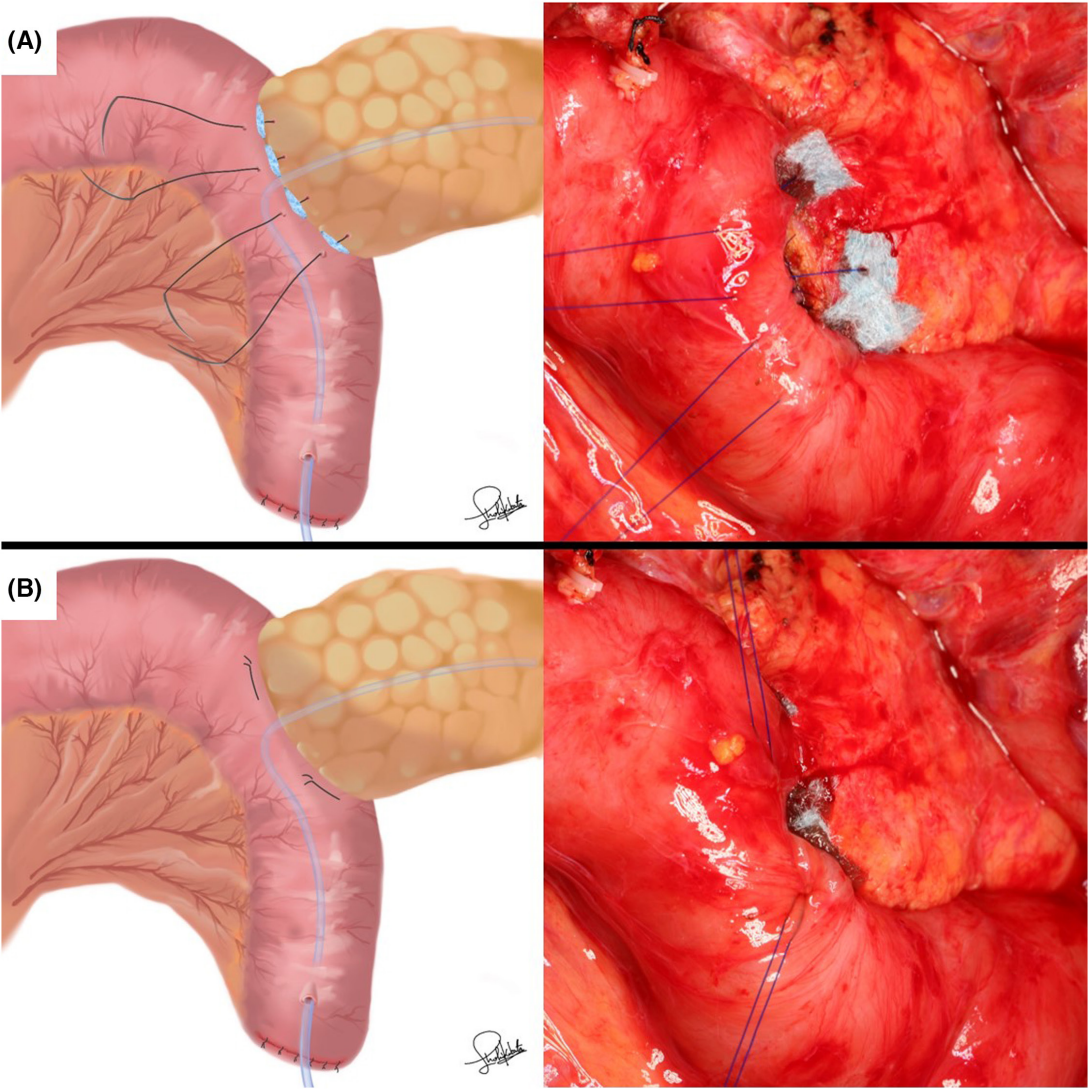
(A) The seromuscular layer of the jejunum was threaded through by the former four straight needles. (B) Each of the two threads was tied

**FIGURE 4:**
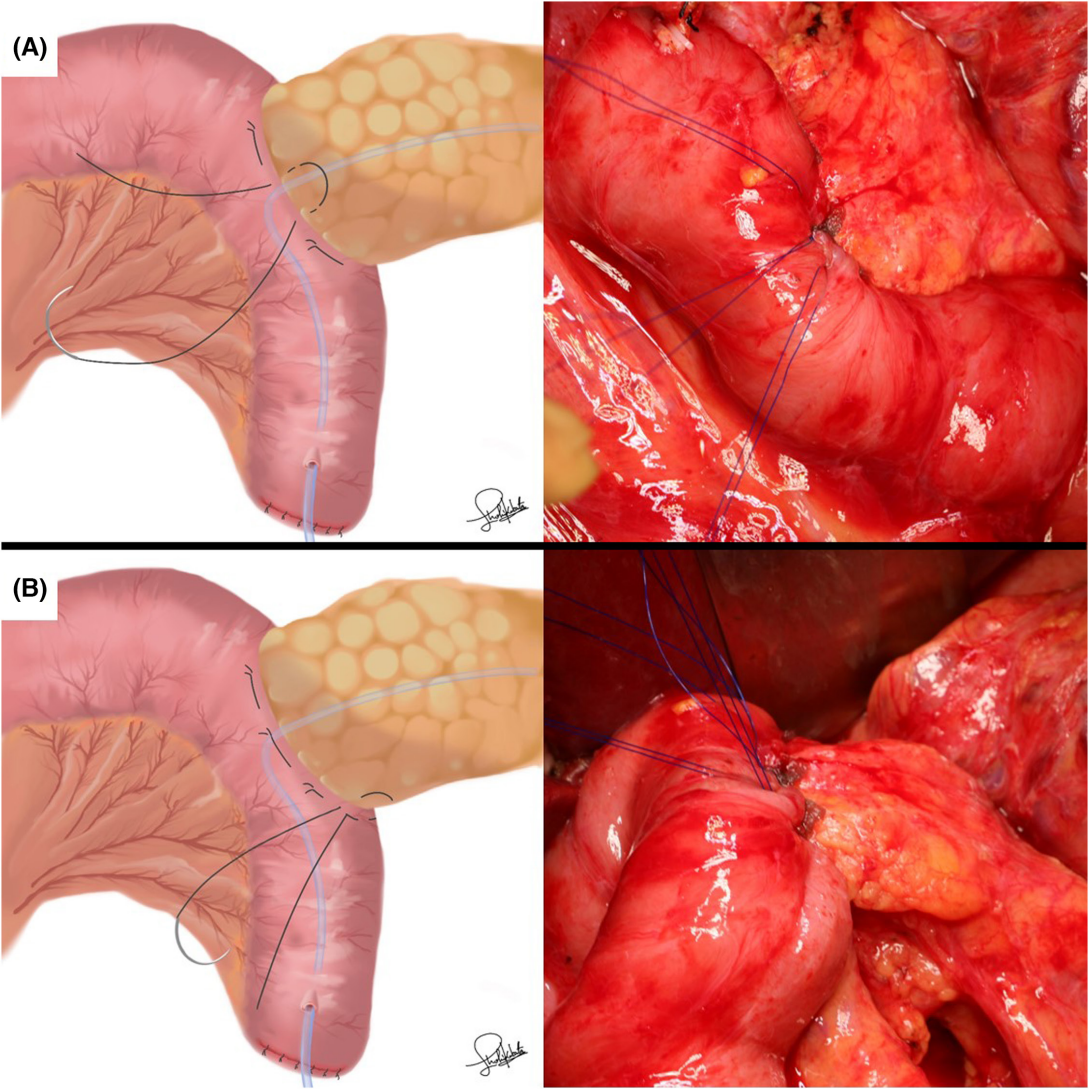
(A) The space between the ligated threads was reinforced with 4‐0 monofilament nonabsorbable sutures. (B) The caudal side was reinforced with 4‐0 monofilament nonabsorbable sutures

In the PGA group, 0.3‐mm‐thick PGA felts (Neoveil; Gunze, Osaka, Japan) were prepared as eight pieces cut into sizes of 7 × 7 mm. After the seromuscular layer of the jejunum was threaded through using straight needles with 4‐0 monofilament nonabsorbable sutures, 7 × 7 mm PGA felts were penetrated by each needle to reinforce the dorsal side (Figure 1A). The pancreatic parenchyma was sutured penetratingly (Figure 1B). For reinforcement of the ventral side, 7 × 7 mm PGA felts were penetrated by each needle (Figure 2A). Anastomosis between the main pancreatic duct and the jejunal wall was performed with 6‐8 interrupted 5‐0 monofilament absorbable sutures (Figure 2B). The seromuscular layer of the jejunum was threaded through with the former four straight needles (Figure 3A). Each of the two threads was tied (Figure 3B). The space between the ligated threads was reinforced with 4‐0 monofilament nonabsorbable sutures (Figure 4A). Next, the caudal side was reinforced with 4‐0 monofilament nonabsorbable sutures (Figure 4B).

Application of fibrin glue around the anastomotic site and the administration of somatostatin analogs were not used in either group.

In this study, period laparoscopic PD was performed in two patients (with PGA group). However, pancreatojejunostomy of both patients was performed in the same manner as above under a small laparotomy.

The drain was removed on postoperative d (PODs) 4 or 5 if the drainage fluid was clear and pancreatic fistula and bacterial contamination were absent. In the case of bacterial contamination detected based on a bacterial culture test of the drain fluid or suspected by the nonserous (turbid) fluid of the drain, drains were exchanged on POD 7. Intraabdominal abscess was defined as a fluid collection by computed tomography (CT), and requiring antibacterial drug or drainage in patients with fever increase.

### 2.2 Statistical analysis

Continuous data are expressed as the medians (ranges). The statistical analyses were performed using chi‐square tests, Mann–Whitney *U* tests, or Fisher's exact probability tests, as appropriate. To overcome biases owing to the different distribution of covariates among patients who underwent Blumgart anastomosis with PGA and without PGA, propensity score matching was carried out. Using multivariate logistic regression analysis, we estimated propensity scores for these patients. The following covariates were selected by the forward selection method from age, sex, preoperative serum albumin concentration, body mass index (BMI), pancreatic ductal adenocarcinoma (PDAC), pancreatic duct size, and pancreatic texture, as these variables were shown to be prognostically significant in other studies. Subsequently, a one‐to‐one match between the two groups was performed by the nearest‐neighbor matching method within 0.05. The balance of covariates between the groups was assessed by the absolute standardized mean difference (ASMD) before and after the matching procedure.

The variables identified as potentially significant by univariate analysis were selected for multivariate analysis with a logistic regression model to identify the independent predictors of CR‐POPF. All *P* values were two‐sided, and *P <* .05 was considered to indicate a statistically significant difference. All statistical calculations were performed using the IBM SPSS Statistics 27 software package (IBM, Tokyo, Japan).

## 3 RESULTS

### 3.1 Propensity score matching analysis

The patient flow chart, including the propensity score matching analysis, is outlined in Figure S1. Thirty‐five (35.7%) of the 98 patients in the without‐PGA group were matched with an equal number from the with‐PGA group after adjusting for covariates.

### 3.2 Patient characteristics

The patient demographics and clinical characteristics are shown in Table S1. Before propensity score matching, the percentages of age (≥70 y), sex, BMI (≥24 kg/m^2^), PDAC, pancreatic duct size (<3 mm), and soft pancreatic texture did not differ between the two groups. However, the percentage of serum albumin (<3.5 g/dl) was significantly higher in the without‐PGA group than in the with‐PGA group (41% vs 17%, *P* = .013).

After propensity score matching, the percentages of age (≥70 y), sex, serum albumin (<3.5 g/dl), BMI (≥24 kg/m^2^), PDAC, pancreatic duct size (<3 mm), and soft pancreatic texture did not differ between the two groups.

### 3.3 Surgical outcomes

Table 1 shows the surgical outcomes after Blumgart anastomosis with/without PGA. After propensity score matching, the operation time was significantly longer in the without‐PGA group than in the with‐PGA group (441 min vs 377 min, *P* = .023). The total blood loss volume, portal vein resection rate, and drain fluid amylase concentration (DFAC) d 1 did not differ between the two groups. DFAC d 3 in the with‐PGA group was one‐third lower than that in the without‐PGA group, but there was no significant difference.

**TABLE 1:** Surgical outcomes of patients who underwent Blumgart anastomosis with/without PGA before and after propensity score matching

	Before propensity matching			After propensity matching		
	Without PGA (n = 98)	With PGA (n = 35)	*P*	Without PGA (n = 35)	With PGA (n = 35)	*P*
Operation time (min)	413 (260–660)	377 (246–771)	.268	441 (294–598)	377 (246–771)	.023
Total blood loss (mL)	457 (65–3170)	450 (20–1965)	.798	502 (65–1931)	450 (20–1965)	.553
Portal vein resection	16 (16%)	6 (17%)	1.000	7 (20%)	6 (17%)	1.000
DFAC‐d1 (U/L)	740 (11–32 048)	1759 (17–13 829)	.978	1844 (11–19 431)	1759 (17–13 829)	.301
DFAC‐d3 (U/L)	454 (8–21 577)	327 (9–25 927)	.822	1063 (9–6506)	327 (9–25 927)	.296
Morbidity						
CR‐POPF	33 (34%)	2 (6%)	.001	17 (49%)	2 (6%)	<.001
Clavien grade 2/3a/3b/4	0/32/1/0	0/2/0/0		0/17/0/0	0/2/0/0	
Bile leakage	4 (4%)	1 (3%)	1.000	0	1 (3%)	1.000
Clavien grade 2/3a/3b/4	1/1/1/1	0/1/0/0		―	0/1/0/0	
Intraabdominal abscess	8 (8%)	8 (23%)	.033	3 (9%)	8 (23%)	.188
Clavien grade 2/3a/3b/4	5/3/0/0	5/3/0/0		1/2/0/0	5/3/0/0	
Intraabdominal bleeding	1 (1%)	0	1.000	0	0	―
Clavien grade 2/3a/3b/4	0/1/0/0	―		―	―	
Delayed gastric emptying	5 (5%)	4 (11%)	.242	3 (9%)	4 (11%)	1.000
Clavien grade 2/3a/3b/4	5/0/0/0	4/0/0/0		3/0/0/0	4/0/0/0	
Septicemia	2 (2%)	0	1.000	1 (3%)	0	1.000
Clavien grade 2/3a/3b/4	0/1/0/1	―		0/1/0/0	―	
Postoperative hospital stays	18 (8–138)	14 (8–104)	.090	21 (8–77)	14 (8–104)	.026
Readmission	24 (24%)	8 (23%)	1.000	7 (20%)	8 (23%)	1.000
Mortality	0	0	―	0	0	―

*Note*: Expressed as N (%) or median (range).

Abbreviations: BMI, body mass index; CR‐POPF, clinically relevant postoperative pancreatic fistula; DFAC, drain fluid amylase concentration; PGA, polyglycolic acid.

CR‐POPF was found in 17 patients (49%) in the without‐PGA group and two patients (6%) in the with‐PGA group (*P* < .001). Among the CR‐POPF patients, all patients had grade B POPF. There were no significant differences between the two groups in terms of bile leakage (0% in the without‐PGA group vs 3% in the with‐PGA group), intraabdominal abscess (9% in the without‐PGA group vs 23% in the with‐PGA group), intraabdominal bleeding (0% in the without‐PGA group vs 0% in the with‐PGA group), DGE (9% in the without‐PGA group vs 11% in the with‐PGA group), or septicemia (3% in the without‐PGA group vs 0% in the with‐PGA group). The number of patients who required abscess drainage insertion were two patients (6%) in the without‐PGA group and three patients (9%) in the with‐PGA group. The postoperative hospital stay was significantly shorter in the with‐PGA group than in the without‐PGA group (14 dd vs 21 dd, *P* = .026). The readmission rate did not differ between the two groups (20% in the without‐PGA group vs 23% in the with‐PGA group). The in‐hospital and 90‐‐d postoperative mortality rates were zero in both groups.

To eliminate the bias due to the learning curve of the Blumgart anastomosis, in the second half (n = 49) of the without‐PGA group and in the with‐PGA group (n = 35) were compared. The median operation time was well adjusted in both groups (378 min vs 377 min, *P* = .443). Nevertheless, the rate of CR‐POPF was significantly lower in the with‐PGA group than in the second half of without‐PGA group (6% vs 24%, *P* = .035).

### 3.4 Predictors of CR‐POPF after Blumgart anastomosis

The multivariate analysis results indicated that male sex (odds ratio [OR]: 3.45, 95% CI: 1.25–9.46, *P* = .016), pancreatic duct size <3 mm (OR: 4.84, 95% CI: 1.74–13.51, *P* = .003), soft pancreatic texture (OR: 3.90, 95% CI: 1.20–12.64, *P* = .023), and no use of PGA (OR: 12.49, 95% CI: 2.59–60.27, *P* = .002) were independently associated with CR‐POPF after PD (Table S2).

## 4 DISCUSSION

Our new penetrating method, like previous methods, was developed with the concept of reinforcing the pancreatic parenchyma from injury caused by the suture. In addition, our PGA felt fills and reinforces the jejunum and pancreas. Furthermore, reinforcing the space between the ligated threads (Figure 4A) and the caudal side (Figure 4B) of the Blumgart anastomosis improves the close contact of the anastomosis and prevents pancreatic juice leakage. Our method is expected to be able to adhere to the anastomotic site for a longer time than the wrapping method. Consequently, in this study using propensity score analysis, the rate of CR‐POPF was significantly lower in the with‐PGA group (6%) than in the without‐PGA group (49%) (*P* < .001). Furthermore, according to multivariate analysis, nonapplication of PGA felt was an independent predictor of CR‐POPF after Blumgart anastomosis. Additionally, DFAC‐d 3 in the with‐PGA group was one‐third lower than that in the without‐PGA group. There were no significant differences between the two groups in terms of intraabdominal abscess. However, the rate of intraabdominal abscess tended to be higher in the with‐PGA group than in the without‐PGA group. Since our drain‐management strategy has not changed,^12^ the reason for the increase in intraabdominal abscess may be due to latent POPF after using PGA felt.

Although there have been remarkable recent advances in pancreatic surgery, CR‐POPF still occurs at a high rate.^2^, ^3^ In addition to surgical methods and instruments, various additional substances have been used to reduce the incidence of CR‐POPF after PD. Fibrin glue may be used to reduce the incidence of CR‐POPF in pancreatic anastomosis. However, a recent randomized control trial and meta‐analysis that evaluated the use of fibrin glue in pancreatic surgery showed no significant effect.^13^ Application of fibrin glue around the anastomotic site and administration of somatostatin analogs were not employed in this study. As an additional substance, PGA has attracted attention in recent years, and PGA felt has been used to prevent POPF in pancreatic surgery.^6^, ^7^, ^8^, ^9^, ^10^

PGA felt is a bioabsorbable tissue‐reinforcing material that is made of PGA and does not remain as a foreign substance in the human body. When PGA felt is attached to a tissue, inflammatory cells and fibroblasts gather around the PGA fibers as a biological reaction; these cells then infiltrate the felt and proliferate, with consequent formation of granulation tissue. The mechanism of reinforcement is that the granulation tissue becomes fibrotic with decomposition of the PGA felt and is replaced with autologous tissue.

Regarding distal pancreatectomy, Jang et al^6^ demonstrated in a randomized controlled trial that application of PGA felt to the cut surface of the pancreas is associated with a significantly reduced rate of CR‐POPF. In that study, the rate of CR‐POPF was 11.4% in the PGA group and 28.3% in the control group (*P* = .04). For PD, there are only a few retrospective studies of CR‐POPF after PD with PGA. Ochiai et al^7^ reported that CR‐POPF occurred in 5.6% of the PGA group and 38.9% of the control group (*P* = .016). Kang et al^8^ reported that the rate of CR‐POPF was significantly lower in the PGA group (12.6%) than in the control group (22.4%) (*P* = .024). On the other hand, some other studies did not show a significant difference in the incidence of CR‐POPF between patients with and without PGA.^9^, ^10^ Although the Blumgart anastomosis has been modified in various institutions with good outcomes, the pancreaticojejunostomy in these studies was not performed via Blumgart anastomosis. Moreover, in these studies the PGA felt was wrapped around the anastomotic site as a method of reinforcement. The wrapping method provides reinforcement on the pancreatic side; however, when a pancreatic fistula develops from the anastomotic site, the reinforcement by the PGA felt gradually weakens, and a space may occur between the PGA felt and the anastomotic site. Therefore, considering this background, we developed a new method by which the PGA felt can adhere without wrapping during Blumgart anastomosis.

This investigation has some limitations that should be mentioned. First, it was a single‐center retrospective study. However, the strength of this study is that the surgical techniques and postoperative management were unified. In addition, propensity score matching controlled for possible selection biases. Second, the rate of CR‐POPF after PD was higher than that in previous studies. It is possible that a learning curve was present in the Blumgart anastomosis of this study. The rationale is that the operation time was significantly longer in the without‐PGA group than in the with‐PGA group. In the future, large‐series multicenter prospective studies evaluating the clinical impact of PGA felt will help to compensate for the limitations of this investigation.

In conclusion, the results of our study indicate that Blumgart anastomosis with our new penetrating method for PGA felt reduces the incidence of CR‐POPF after PD. We therefore propose the application of PGA felt by the penetrating method to prevent CR‐POPF after PD.

## DISCLOSURE

Funding: The author(s) received no financial support for the research, authorship, and/or publication of this article.

Conflict of Interest: The authors have no conflicts of interest to disclose.

Ethics Approval and Consent to Participate: This study was approved by the Institutional Review Board of Aichi Medical University (No. 2021‐149) and performed in accordance with the 1964 Declaration of Helsinki and its later amendments or comparable ethical standards. All patients signed informed consent forms before surgery.

Author Contributions: Fukami designed the study and wrote the initial draft of the article. Sano contributed to interpretation of the data and critical revision of the article for important intellectual content. All the other authors (TS, TO, KY, KS, SK, TM, SK, and KK) contributed to the data collection and interpretation and critically reviewed the article. All the authors have read and approved the final version of the article and have agreed to be accountable for all aspects of the study, ensuring that any questions related to the accuracy or integrity of any part of the work are resolved.

## Supporting information


Table S1

Table S2

Figure S1
