# Supplementary material for: Blumgart anastomosis with polyglycolic acid felt reduces the incidence of pancreatic fistula after pancreaticoduodenectomy: A propensity score analysis
Source: Ann Gastroenterol Surg. 2022 Jun 30;6(6):880–6. doi: 10.1002/ags3.12598 (PMC9628387; doi:10.1002/ags3.12598)
Supplement: Supplementary file 1 — Table S1 [file AGS3-6-880-s001.docx]

**Supplementary Table 1. Characteristics of patients who underwent Blumgart anastomosis with/without PGA**

**before and after propensity score matching**

|  | Before Propensity Matching | | | | | | | |  | After Propensity Matching | | | | | | | |
| --- | --- | --- | --- | --- | --- | --- | --- | --- | --- | --- | --- | --- | --- | --- | --- | --- | --- |
|  | Without PGA (n=98) | |  | With PGA (n=35) | |  | *P* | ASMD |  | Without PGA (n=35) | |  | With PGA (n=35) | |  | *P* | ASMD |
| Variables | N | % |  | N | % |  |  |  |  | N | % |  | N | % |  |  |  |
| Age 70 > years | 55 | 56% |  | 19 | 54% |  | 0.851 | 0.02 |  | 19 | 54% |  | 19 | 54% |  | 1.000 | 0.00 |
| Sex Male | 57 | 58% |  | 22 | 63% |  | 0.627 | 0.04 |  | 12 | 34% |  | 13 | 37% |  | 0.803 | 0.03 |
| Serum albumin < 3.5 g/dL | 40 | 41% |  | 6 | 17% |  | 0.013 | 0.22 |  | 7 | 20% |  | 6 | 17% |  | 1.000 | 0.04 |
| BMI > 24 kg/m^2^ | 25 | 26% |  | 9 | 26% |  | 1.000 | 0.01 |  | 13 | 37% |  | 9 | 26% |  | 0.440 | 0.12 |
| PDAC | 54 | 55% |  | 16 | 46% |  | 0.340 | 0.08 |  | 14 | 40% |  | 16 | 46% |  | 0.629 | 0.06 |
| Pancreatic duct size < 3 mm | 46 | 47% |  | 16 | 46% |  | 0.901 | 0.01 |  | 15 | 43% |  | 16 | 46% |  | 0.810 | 0.03 |
| Soft pancreatic texture | 59 | 60% |  | 24 | 69% |  | 0.380 | 0.08 |  | 23 | 66% |  | 24 | 69% |  | 0.799 | 0.03 |

PGA, polyglycolic acid; BMI, body mass index;

PDAC, pancreatic ductal adenocarcinoma;

ASMD, absolute standardized mean difference
