# Supplementary material for: Blumgart anastomosis with polyglycolic acid felt reduces the incidence of pancreatic fistula after pancreaticoduodenectomy: A propensity score analysis
Source: Ann Gastroenterol Surg. 2022 Jun 30;6(6):880–6. doi: 10.1002/ags3.12598 (PMC9628387; doi:10.1002/ags3.12598)
Supplement: Supplementary file 2 — Table S2 [file AGS3-6-880-s002.docx]

**Supplementary Table 2. Uni- and multivariate predictors of CR-POPF after Blumgart anastomosis (n=133)**

|  | | Univariate | | | Multivariate | | |
| --- | --- | --- | --- | --- | --- | --- | --- |
| Variable | | OR | 95% CI | *P* | OR | 95% CI | *P* |
| Age (years) | |  |  |  |  |  |  |
|  | > 70 (n=74) | 1.09 | 0.40-2.96 | 0.863 |  |  |  |
|  | < 70 (n=59) | 1 |  |  |  |  |  |
| Sex | |  |  |  |  |  |  |
|  | Male (n=79) | 3.22 | 1.08-9.62 | 0.036 | 3.45 | 1.25-9.46 | 0.016 |
|  | Female (n=54) | 1 |  |  | 1 |  |  |
| Serum albumin (g/dL) | |  |  |  |  |  |  |
|  | < 3.5 (n=46) | 0.48 | 0.16-1.47 | 0.199 |  |  |  |
|  | > 3.5 (n=87) | 1 |  |  |  |  |  |
| BMI (kg/m^2^) | |  |  |  |  |  |  |
|  | > 24 (n=34) | 1.61 | 0.55-4.68 | 0.382 |  |  |  |
|  | < 24 (n=99) | 1 |  |  |  |  |  |
| Operation time (min) | |  |  |  |  |  |  |
|  | > 480 (n=31) | 0.93 | 0.28-3.09 | 0.900 |  |  |  |
|  | < 480 (n=102) | 1 |  |  |  |  |  |
| Total blood loss (mL) | |  |  |  |  |  |  |
|  | > 1,000 (n=18) | 1.69 | 0.37-7.67 | 0.500 |  |  |  |
|  | < 1,000 (n=115) | 1 |  |  |  |  |  |
| Pancreatic duct size (mm) | |  |  |  |  |  |  |
|  | < 3 (n=62) | 5.54 | 1.81-16.92 | 0.003 | 4.84 | 1.74-13.51 | 0.003 |
|  | > 3 (n=71) | 1 |  |  | 1 |  |  |
| Pancreatic texture | |  |  |  |  |  |  |
|  | Soft (n=83) | 4.06 | 1.11-14.92 | 0.035 | 3.90 | 1.20-12.64 | 0.023 |
|  | Hard (n=50) | 1 |  |  | 1 |  |  |
| PGA | |  |  |  |  |  |  |
|  | Without (n=98) | 15.43 | 3.02-78.79 | 0.001 | 12.49 | 2.59-60.27 | 0.002 |
|  | With (n=35) | 1 |  |  | 1 |  |  |

CR-POPF, clinically relevant postoperative pancreatic fistula;

BMI, body mass index;

PGA, polyglycolic acid
