# Supplementary figures and images for: Blumgart anastomosis with polyglycolic acid felt reduces the incidence of pancreatic fistula after pancreaticoduodenectomy: A propensity score analysis
Source: Ann Gastroenterol Surg. 2022 Jun 30;6(6):880–6. doi: 10.1002/ags3.12598 (PMC9628387; doi:10.1002/ags3.12598)

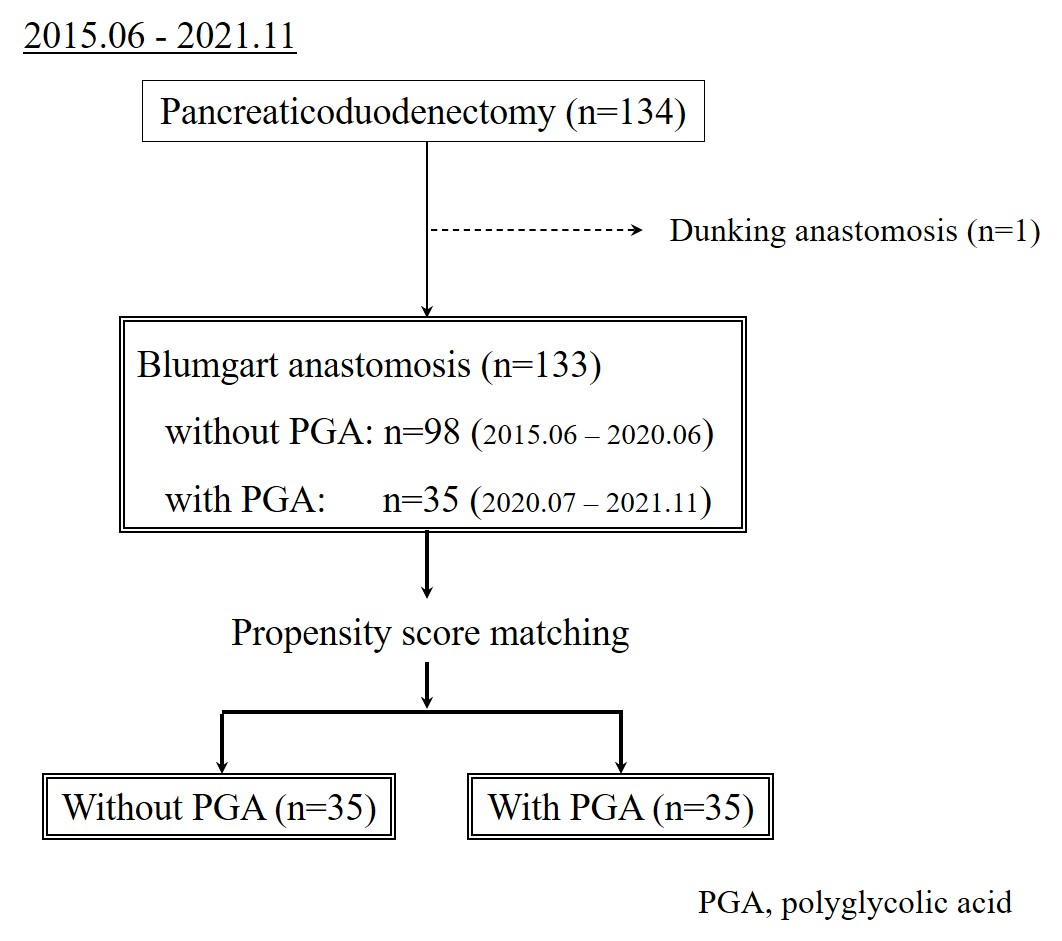

Supplement: Supplementary file 3 — Figure S1 [file AGS3-6-880-s003.tiff]
